# Influence of polyvascular disease on clinical outcome in patients undergoing transcatheter aortic valve implantation via transfemoral access

**DOI:** 10.1371/journal.pone.0260385

**Published:** 2021-12-02

**Authors:** Masahiro Yamawaki, Yosuke Honda, Kenji Makino, Takahide Nakano, Yasunori Iida, Fumiaki Yashima, Hiroshi Ueno, Kazuki Mizutani, Minoru Tabata, Norio Tada, Kensuke Takagi, Futoshi Yamanaka, Toru Naganuma, Yusuke Watanabe, Masanori Yamamoto, Shinichi Shirai, Kentaro Hayashida, on behalf of OCEAN-TAVI registry

**Affiliations:** 1 Department of Cardiology, Saiseikai Yokohama City Eastern Hospital, Yokohama, Japan; 2 Department of Cardiovascular Surgery, Saiseikai Yokohama City Eastern Hospital, Yokohama, Japan; 3 Department of Cardiology, Saiseikai Utsunomia Hospital, Utsunomia, Japan; 4 Second Department of Internal Medicine, University of Toyama, Toyama, Japan; 5 Department of Cardiovascular Medicine, Osaka City University Graduate School of Medicine, Osaka, Japan; 6 Department of Cardiovascular Surgery, Tokyo Bay Urayasu Ichikawa Medical Center, Urayasu, Japan; 7 Department of Cardiology, Sendai Kousei Hospital, Sendai, Japan; 8 Department of Cardiology, Ogaki Municipal Hospital, Ogaki, Japan; 9 Department of Cardiology, Shonan Kamakura General Hospital, Kamakura, Japan; 10 Department of Cardiology, New Tokyo Hospital, Matsudo, Japan; 11 Department of Cardiology, Teikyo University School of Medicine, Tokyo, Japan; 12 Department of Cardiology, Toyohashi Heart Center, Toyohashi, Japan; 13 Department of Cardiology, Nagoya Heart Center, Nagoya, Japan; 14 Department of Cardiology, Kokura Memorial Hospital, Kitakyushu, Japan; 15 Department of Cardiology, Keio University School of Medicine, Tokyo, Japan; Baylor Scott and White, Texas A&M College of Medicine, UNITED STATES

## Abstract

**Background:**

The influence of polyvascular disease (PVD) on the short- and long-term clinical outcomes of patients undergoing transcatheter aortic valve implantation via trans-femoral access (TF-TAVI) has not been fully elucidated.

**Methods:**

A total of 2167 patients from the Optimized CathEter vAlvular iNtervention-TAVI (OCEAN-TAVI) registry who underwent TF-TAVI was studied. PVD was defined as the presence of at least two of the following vascular bed (VB) diseases: concomitant coronary artery disease (CAD), cerebrovascular disease (CVD), and peripheral artery disease (PAD).

**Results:**

Patients with PVD (288 patients, 13.3%) had a higher incidence of in-hospital complications, such as AKI (16.3% vs. 7.0%, p<0.01) and disabling stroke (3.5% vs. 1.2%, p<0.01) than patients without PVD. These complications caused higher rates of procedural mortality (4.5% vs. 2.0%, p<0.01). PVD increased the risk of the 2-year rate of cardiovascular death (adjusted hazard ratio [HR], 1.61; 95% confidence interval [CI], 1.04–2.50; p<0.05); however, non-cardiovascular death, myocardial infarction, or ischemic stroke was not associated with PVD. Worsening heart failure (4.6% vs. 1.1%, p<0.01) was the main cause of cardiovascular death among patients with PVD. In a sub-analysis, compared with patients with AS alone, those with 2 VB diseases (CAD+PAD; adjusted HR, 1.93; 95% CI, 1.06–3.53; p<0.05) and 3 VB diseases (CAD+CVD+PAD; adjusted HR, 2.61; 95% CI, 1.21–5.62; p<0.05) had a higher risk of 2-year cardiovascular death.

**Conclusions:**

The increased prevalence of concomitant atherosclerotic VB diseases before TF-TAVI may increase the rates of in-hospital complications and 2-year cardiovascular death. Given the higher rate of mortality in patients with PVD undergoing TF-TAVI, future studies focusing on medical therapy are needed to reduce long-term cardiovascular events in this high-risk subset.

## Introduction

Transcatheter aortic valve implantation (TAVI) is the treatment of choice for patients with symptomatic severe aortic valve stenosis (AS) who are deemed inoperative or at a high surgical risk [1]. Comorbid conditions and frailty in routine TAVI clinical practice can affect short- and long-term outcomes. After the recent results from the PARTNER-3 and EVOLUT low-risk trials [2, 3], the indication for TAVI is expected to expand to low-risk patients. Furthermore, in light of the evidence on the long-term durability of TAVI and the corresponding improved survival, it is important to study long-term cardiac events and strategies for optimal management [4].

Risk factors for AS are similar to those for atherosclerosis. Consequently, concomitant vascular bed (VB) diseases, such as coronary artery disease (CAD), peripheral arterial disease (PAD), and cerebrovascular disease (CVD), are often identified before the procedure.

The Reduction of Atherothrombosis for Continued Health (REACH) registry showed that patients with prior ischemic events have a higher rate of subsequent ischemic event [5]. In the literature, patients with polyvascular disease (PVD) have the highest risk of long-term cardiovascular events, and this has been described in many other cardiovascular studies and in the European Society of Cardiology (ESC) guidelines [5–9].

The TAVI population includes patients at high surgical risk, with extremely old, frail, and inoperative patients being the most common. Furthermore, although the influence of CAD, PAD, or CVD on patients undergoing TAVI has been studied [10–12], the effect of PVD and combination of these VBs disease (i.e. CAD+PAD, CAD+CVD, PAD+CAD, CAD+PAD+CVD) on clinical outcome are scarcely investigated. Herein, we elucidated the effect of PVD on the short- and long-term clinical outcomes of patients with severe AS undergoing transfemoral TAVI.

## Materials and methods

### Study subjects

Data were collected from the Optimized CathEter vAlvular iNtervention-TAVI registry (OCEAN-TAVI; UMIN000020423, https://ocean-shd.com). This is an ongoing prospective multi-institutional registry study conducted at 14 institutions in Japan. Of the 2588 all-comer consecutive patients with severe AS undergoing TAVI between October 2013 and May 2017, 421 patients treated via an alternative access approach (transapical, 357 patients; direct aortic, 15 patients; trans-subclavian, 19 patients; and trans-iliac, 30 patients) were excluded. We finally enrolled 2167 patients undergoing TAVI via a transfemoral approach. This study adheres to the principles of the Declaration of Helsinki, and the study protocol was approved by the medical ethics committee of all institutions. Informed consent was obtained from all patients.

### Definition

PVD and the general diagnostic criteria for each disease territory were defined according to a previous review of PVD and the REACH registry [5, 13]. Patients were diagnosed with CAD if one of the following criteria was present: (i) prior coronary revascularization, (ii) stable angina, and (iii) prior myocardial infarction (MI) or unstable angina [5, 13]. In this study, scheduled percutaneous coronary intervention (PCI) before and during TAVI was included in prior coronary revascularization.

Patients were diagnosed with peripheral artery disease (PAD) if one of the following criteria was present: (i) intermittent claudication with documented lower artery stenosis/occlusion, (ii) an ankle brachial pressure index (ABI) < 0.9 in either leg, and (iii) previous interventions for angioplasty, stenting, atherectomy, or peripheral artery bypass grafting, or other vascular interventions, including amputation [5, 13]. ABI was measured by the oscillometric method in 1908 patients (88%) before TAVI using the VaSera series (Fukuda Denshi, Tokyo, Japan) or Form series (Omron Healthcare, Kyoto, Japan).

Patients were diagnosed with CVD if one of the following criteria were present: (i) history of ischemic stroke, transient ischemic attack, carotid endarterectomy, or carotid artery stenting and (ii) carotid artery stenosis (>50%) detected with ultrasound using the method reported in the North American Symptomatic Carotid Endarterectomy Trial [5, 13]. Bilateral carotid artery ultrasonography was performed in 1829 patients (84%).

PVD was defined in the 2017 ESC guidelines as clinically significant atherosclerosis in at least two major artery territories [9]. Most studies on PVD included CAD, CVD, and PAD as at-risk VBs, with aortic and arterial visceral disease significantly less consistently included and/or reported [13]. Therefore, according to a previous review [13], in this study, CAD, CVD, and PAD were considered a component of PVD, and aortic and visceral disease were not included.

Patients were categorized according to the number of atherosclerotic VBs before TAVI as follows: AS alone (AS without known CAD, PAD, or CVD); 1 VB disease: AS+CVD, AS+CAD, and AS+PAD; 2 VB diseases: AS+CVD+PAD, AS+CAD+CVD, and AS+CAD+PAD; and 3 VB diseases: AS+CAD+CVD+PAD. PVD was defined as the presence of 2 or 3 VB diseases: AS+CVD+PAD, AS+CAD+CVD, or AS+CAD+PAD, and AS+CAD+CVD+PAD, respectively.

The clinical frailty scale was measured by a trained medical professional according to the Canadian Study of Health and Aging grading criteria [14]. Before TAVI, anemia was defined as a hemoglobin level of <11 g/dL, and thrombocytopenia was defined as a platelet count <100×10^9^/L [15]. Chronic kidney disease (CKD) stage 4 or 5 was defined as an estimated glomerular filtration rate of <29 mL/min/1.73 m^2^ [15].

Single antiplatelet therapy included aspirin, P2Y12 antagonist, or cilostazol, and dual antiplatelet therapy included two of the following drugs: aspirin, P2Y12 antagonist, and cilostazol. Oral anticoagulation (OAC) was defined as warfarin or direct OAC intake.

### Endpoint

The rate of in-hospital complications was determined. The primary endpoint in this study was cardiovascular death within the first 2 years postoperatively, and secondary endpoints were non-cardiovascular death within the aforementioned 2 years, all-cause death, ischemic stroke, myocardial infarction (MI), major or life-threatening bleeding, and hospital readmission after TAVI.

According to on-site reports, the cause of death was determined and classified into any of the following modified Definition of Valve Academic Research Consortium-2 criteria [16]: (1) procedural mortality; (2) MI; (3) worsening heart failure (HF); (4) non-coronary vascular death, i.e., pulmonary embolism, ruptured aortic aneurysm, dissecting aneurysm, gastrointestinal ischemia, lower extremity ischemia, or other vascular diseases; (5) stroke; (6) valve-related deaths, such as structured or non-structured valve dysfunction, i.e., infected endocarditis; (7) sudden or unwitnessed death; (8) unknown or other causes of cardiovascular death; and (9) non-cardiovascular death. Procedural mortality comprised all-cause deaths within 30 days or during index procedure hospitalization if the postoperative length of stay was longer than 30 days [16], valve-related deaths included structural or non-structural valve dysfunctions or other valve-related adverse events, strokes included ischemic or hemorrhagic strokes, and non-coronary vascular deaths did not include strokes in this study.

### Statistical analysis

Continuous variables are presented as mean ± standard deviation (SD), and dichotomous variables are presented as numbers and percentages. We performed the Pearson bivariate analysis, chi-square test, and Fisher’s exact test for categorical analysis between groups. An unpaired t-test or Mann–Whitney U test was used for continuous variables. The Kaplan–Meier survival method was used to compare endpoints between groups by the log-rank test. Univariate and multivariate analyses were performed using a Cox proportional-hazards model to determine the adjusted risk for clinical outcomes except for MI because of the low endpoint count of MI (2 patients (0.09%)). Accordingly, the 47 clinically relevant predictors displayed in Table 1, comprising baseline clinical backgrounds, echocardiography data, medication, and procedure, were entered in the univariate analysis. All factors consistent with previous reports as predictors for endpoints [12, 15, 17–25] having P-values of <0.01 were entered to the multivariate analysis. P-values of <0.05 were considered statistically significant. SPSS version 21.0 (Chicago, IL, USA) was used for statistical analysis.

**Table 1:** Patient background, medical therapy at discharge, baseline transthoracic echocardiography findings, and procedural characteristics.

	Patients with PVD (n = 288)	Patients without PVD (n = 1879)	p-value
Male* # ¶	110 (38.2)	531 (28.3)	<0.01
Age, years *	85±5	84±5	0.31
Body mass index * ¶	22.8±3.7	22.2±3.6	<0.01
Clinical frailty scale			
1	2 (0.7)	25 (1.3)	
2	12 (4.2)	130 (6.9)	
3	68 (23.6)	636 (33.8)	
4	98 (34.0)	630 (33.5)	<0.01
5	56 (19.4)	254 (13.5)	
6	38 (13.2)	148 (7.9)	
7	12 (4.2)	53 (2.8)	
8	2 (0.7)	3 (0.2)	
Average of CFS * # ¶ £ @	4.3±1.3	3.9±1.2	<0.01
STS *	11.3±11.0	7.6±5.9	<0.01
Concomitant CAD	259 (89.9)	485 (25.8)	<0.01
Concomitant CVD	150 (52.1)	131 (7.0)	<0.01
Concomitant PAD	217 (75.3)	187 (10.0)	<0.01
NYHA 3 or 4 * # ¶ £ @	172 (59.7)	933 (49.7)	<0.01
Anemia * ¶ £	141 (49.0)	805 (42.8)	0.05
Thrombocytopenia* ¶ £	18 (6.3)	120 (6.4)	0.93
Current smoking *	13 (4.5)	41 (2.2)	<0.05
History of hemorrhagic stroke *	2 (0.7)	9 (0.5)	0.44
Dyslipidemia * ¶	148 (51.4)	760 (40.4)	<0.01
Diabetes mellitus * § £	106 (36.8)	362 (19.3)	<0.01
Hypertension *	234 (81.3)	1411 (75.1)	<0.05
History of cancer *	35 (12.2)	187 (10.0)	0.25
Atrial fibrillation * ¶ £	72 (25.0)	390 (20.8)	0.10
CKD stage 4 or 5 * # ¶ § £	56 (19.4)	199 (10.6)	<0.01
COPD*¶£	43 (14.9)	252 (13.4)	0.48
LC or liver disease* ¶ £ @	9 (3.1)	59 (3.1)	0.99
Medication before discharge			
Single antiplatelet therapy*	37 (12.8)	315 (16.8)	0.09
Dual antiplatelet therapy*	164 (56.9)	1017 (54.1)	0.37
Oral anticoagulant therapy*	18 (6.3)	151 (8.0)	0.29
ACE inhibitor/ARB* ¶	158 (54.9)	983 (52.3)	0.42
Beta blocker* £	114 (39.6)	596 (31.7)	<0.01
Calcium channel blocker*	128 (44.4)	788 (41.9)	0.42
Diuretics* ¶	177 (61.5)	983 (52.3)	<0.01
Statin*¶	140 (48.6)	733 (39.0)	<0.01
Steroids* ¶	12 (4.2)	100 (5.3)	0.41
Transthoracic echocardiography			
Aortic valve area index*	0.4±0.1	0.4±0.1	0.20
LVEF*, %, # ¶	56.8±13.2	60.0±12.5	<0.01
Severe or moderate AR*	33 (11.5)	196 (10.4)	0.60
Severe or moderate MR* £	44 (15.3)	201 (10.7)	<0.05
Severe or moderate MS*	6 (2.2)	26 (1.4)	0.22
Severe or moderate TR* # ¶ £	26 (9.0)	151 (8.0)	0.57
Valve selection			
Sapien XT*	133 (46.2)	882 (46.9)	
Sapien 3*	115 (39.9)	724 (38.5)	0.97
Corevalve*	23 (8.0)	155 (8.2)	
Evolut*	17 (5.9)	118 (6.3)	
Valve size			
20 mm*	17 (5.9)	78 (4.2)	
23 mm*	133 (46.2)	974 (51.8)	0.19
26 mm*	111 (38.5)	641 (34.1)	
29 mm*	27 (9.4)	186 (9.9)	
Local anesthesia*	74 (25.7)	527 (28.0)	0.41
Surgical cut-down*	98/287 (34.1)	572/1862 (30.7)	0.32
Pre-dilation*	169 (58.7)	1148 (61.1)	0.43
Post-dilation*	53 (18.4)	421 (22.4)	0.13
Contrast media*, mL	111±56	118±59	<0.05
Fluoroscopic time*, min	23±12	22±11	0.11

Data are n (%). ACE = Angiotensin converting enzyme inhibitors, ARB = angiotensin; AR = aortic regurgitation, CFS = clinical frailty scale, CKD = chronic kidney disease, COPD = chronic obstructive pulmonary disease, LC = liver cirrhosis, LVEF = left ventricle ejection fraction, MR = mitral regurgitation, MS = mitral stenosis, NYHA = New York Heart Association functional classification, PVD = poly-vascular atherosclerotic disease, TR = tricuspid regurgitation., STS = society of thoracic surgeons predicted risk of mortality, Anemia was defined as a hemoglobin level <11 g/dL, and CKD was defined an as an estimated glomerular filtration rate of <30 mL/min/1.73m^2^. *Factors selected for univariate analysis. Potential independent variables finally selected for multivariate analysis of cardiovascular death (#), all- and non-cardiovascular death (¶), stroke (§), hospital readmission (£), and major or life-threaten bleeding (@).

## Results

### Prevalence, baseline characteristics, medication, and procedure backgrounds

Of the 2167 patients undergoing TF-TAVI, 744 patients (34.3%), 281 patients (13.0%), and 404 patients (18.6%) had concomitant CAD, concomitant CVD, and concomitant PAD, respectively. Of 744 patients with CAD, scheduled PCI was performed before TAVI in 263 patients (35.3%), and immediately before valve implantation during TAVI procedure in 11 patients (1.5%). PVD was detected in 288 patients (13.3%).

Compared with patients without PVD, those with PVD were predominantly male and frail and had a higher body mass index and more comorbidities, including a higher society of thoracic surgeons predicted risk of mortality (STS), higher frequency of concomitant CAG, concomitant CVD, concomitant PAD, a New York Heart Association functional classification 3 or 4, current smoking, dyslipidemia (DL), diabetes mellitus (DM), hypertension (HT), and CKD stage 4 or 5 (Table 1).

For medical therapy, beta-blocker, diuretics, and statin were more likely to be administered to patients with PVD than to those without. On transthoracic echocardiography, the left ventricular ejection fraction was lower (56.8% vs. 60.0%, p<0.01), and mitral regurgitation rate was higher (15.3% vs. 10.7%, p<0.05) in patients with PVD than those without. As for procedural findings, rate of surgical cut-down did not differ among groups. The amount of contrast media was lower in patients with PVD than in those without.

### Procedure complication incidence

Table 2 shows the data of in-hospital complications. The incidence of acute kidney injury (AKI: 16.3% vs. 7.0%, *p*<0.001) and disabling stroke (3.5% vs. 1.2%, p<0.01) was significantly higher in patients with PVD than in those without.

**Table 2:** In-hospital complications.

	Patients with PVD (n = 288)	Patients without PVD (n = 1879)	p-value
Acute kidney injury	47 (16.3)	131 (7.0)	<0.01
Disabling stroke	10 (3.5)	23 (1.2)	<0.01
Vascular complication	36 (12.5)	180 (9.6)	0.12
Hemorrhagic stroke	2 (0.7)	4 (0.2)	0.18
Life-threatening bleeding	13 (4.5)	63 (3.4)	0.32
Pacemaker implants	29 (10.1)	169 (9.0)	0.56
New onset atrial fibrillation	8 (2.8)	53 (2.8)	0.95
Cardiac tamponade	5 (1.7)	27 (1.4)	0.42

Data are n (%). PVD=Polyvascular atherosclerotic disease.

### Two-year clinical outcomes

**Fig 1:**
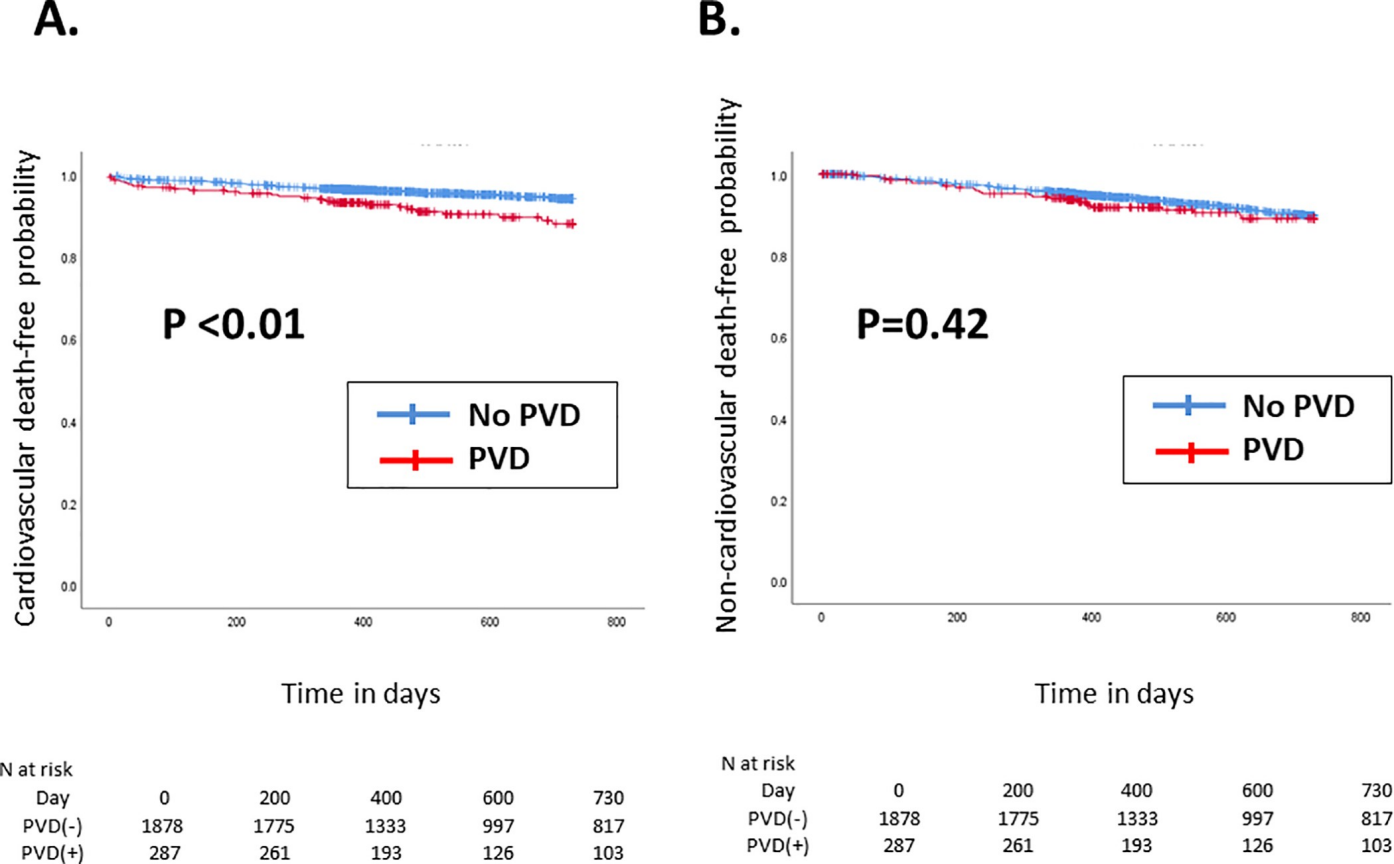
Kaplan–Meier curve of the 2-year (A) cardiovascular death-free and (B) non-cardiovascular death-free rates. The red and blue lines indicate patients with and without PVD, respectively. PVD = polyvascular disease.

**Fig 2:**
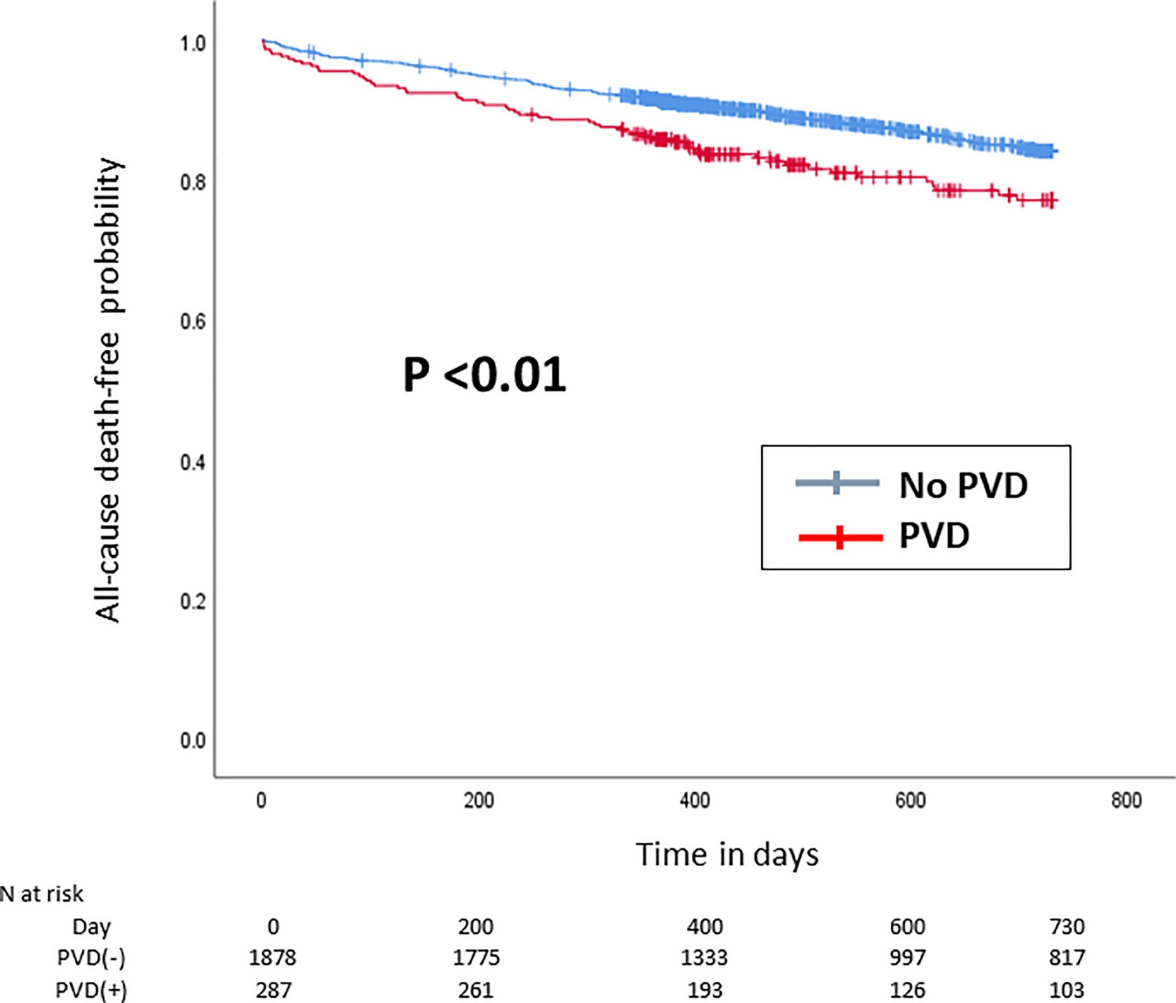
Kaplan–Meier curve of the 2-year (A) all-cause death-free rate. Red and blue lines indicate patients with and without PVD patients, respectively. PVD = polyvascular disease.

**Fig 3:**
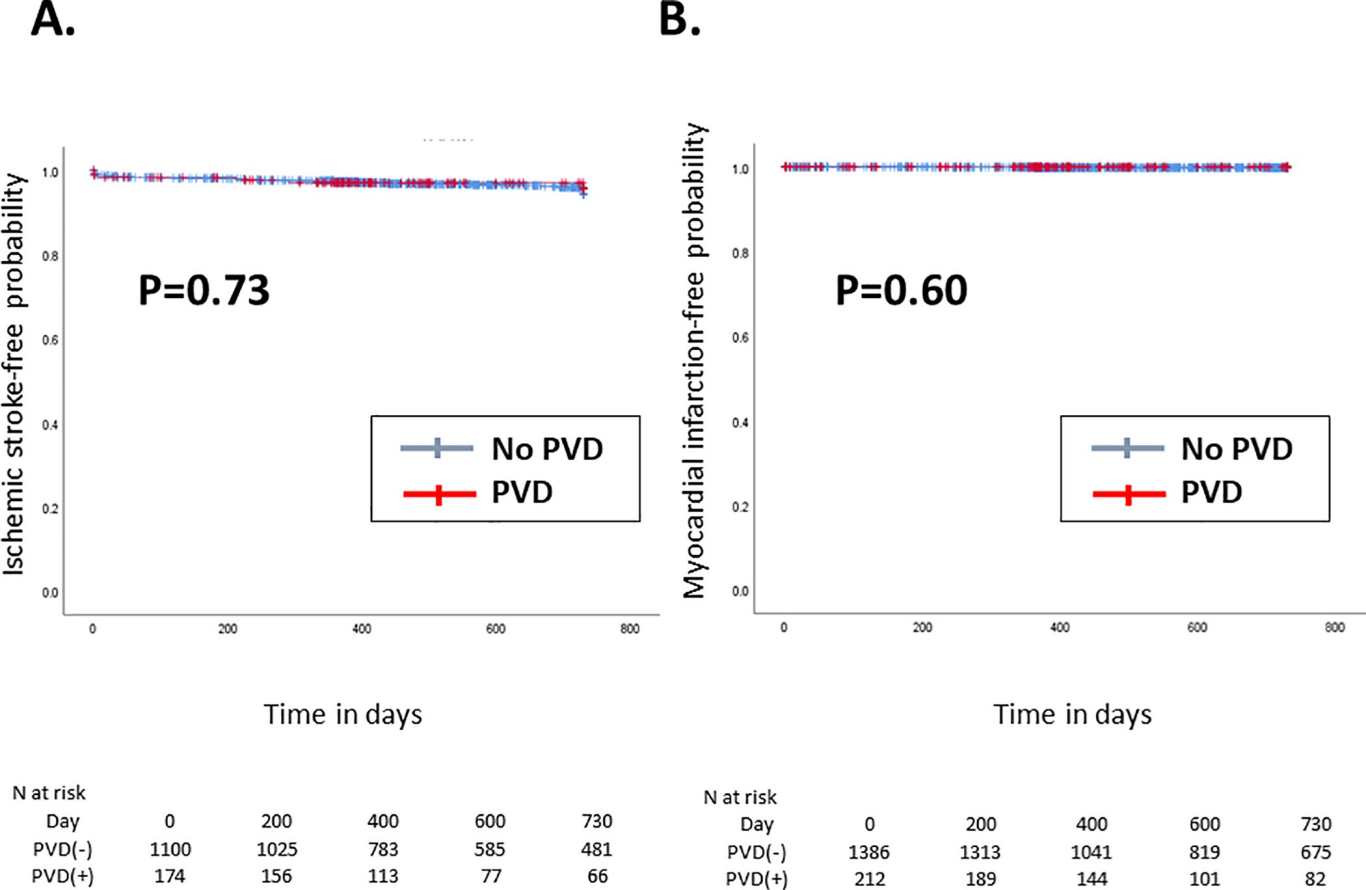
Kaplan–Meier curve of the 2-year (A) ischemic stroke-free and (B) myocardial infarction-free rates. Red and blue lines indicate patients with and without PVD, respectively. PVD = polyvascular disease.

**Fig 4:**
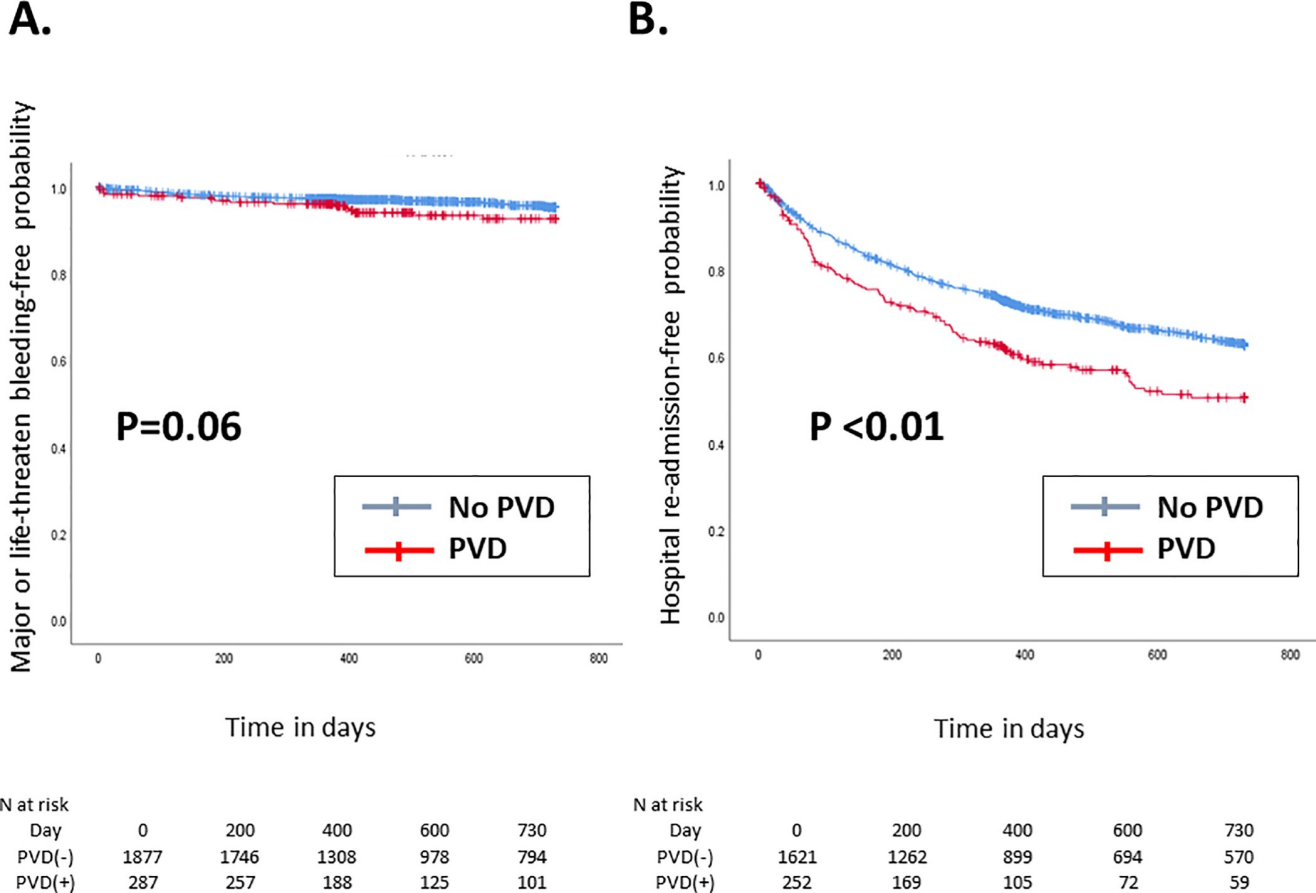
Kaplan–Meier curve of the 2-year (A) major or life-threatening bleeding-free and (B) hospital readmission-free rates. The red and blue lines indicate patients with and without PVD, respectively. PVD = polyvascular disease.

The Kaplan–Meier curve of the 2-year clinical outcomes after TAVI is shown in Figs 1–4.

Compared with patients without PVD, those with PVD had a significantly higher incidence of cardiovascular death (p<0.01, Fig 1A), yet there were no differences in non-cardiovascular death occurrence between patients with PVD and those without (p = 0.42, Fig 1B). Accordingly, the 2-year rate of all-cause death was significantly higher in patients with PVD than those without (p <0.01, Fig 2). In contrast, the incidence of ischemic stroke (*p* = 0.73, Fig 3A) and acute MI (*p* = 0.60, Fig 3B) did not significantly differ between patients with PVD and those without. The 2-year incidence of major or life-threatening bleeding and the incidence of hospital readmission tended to be higher in patients with PVD (p = 0.06 and Fig 4A, and p <0.01 and Fig 4B, respectively).

After the adjustment for confounders by a multivariate analysis, compared with patients without PVD, those with PVD had a higher risk of cardiovascular death within 2 years postoperatively (adjusted hazard ratio [HR], 1.61; 95% confidence interval [CI], 1.04–2.50; p<0.05; Table 3).

**Table 3:** Two-year outcomes of patients with PVD undergoing TAVI.

	Adjusted HR	95% CI	p-value
Cardiovascular death #	1.61	1.04–2.50	<0.05
Non-cardiovascular death ¶	0.97	0.63–1.45	0.87
All-cause death ¶	1.31	0.98–1.76	0.07
Ischemic stroke §	0.69	0.29–1.64	0.40
Hospital readmission £	1.23	0.99–1.52	0.06
Major or life-threaten bleeding @	1.46	0.86–2.48	0.17

CI = confidence interval, HR = hazard ratio, TAVI = transcatheter aortic valve implantation. Other abbreviations are displayed in Table 1.

#, ¶, §, £ and @ indicate variables adjusted for the same confounders in Table 1.

The findings for sub-analysis of adjusted risk for 2-year clinical outcome in AS patients with VB disease compared with AS alone are shown in Table 4.

**Table 4:** Adjusted risk for the 2-year clinical outcomes of patients with AS in addition to CVD, CAD, PAD, CVD+PAD, CAD+CVD, CAD+PAD, and CAD+CVD+PAD undergoing TAVI compared with patients with AS alone.

	Adjusted HR	95% CI	p-value
Cardiovascular death #			
AS+CVD (n = 131)	1.03	0.44–2.40	0.95
AS+CAD (n = 485)	1.07	0.66–1.75	0.78
AS+PAD (n = 187)	1.24	0.65–2.35	0.52
AS+CVD+PAD (n = 29)	0.57	0.08–4.17	0.58
AS+CAD+CVD (n = 71)	1.04	0.37–2.91	0.94
AS+CAD+PAD (n = 138)	1.93	1.06–3.53	<0.05
AS+CAD+CVD+PAD (n = 50)	2.61	1.21–5.62	<0.05
Non-cardiovascular death ¶			
AS+CVD (n = 131)	1.91	1.08–3.38	<0.05
AS+CAD (n = 485)	1.41	0.93–2.13	0.10
AS+ PAD (n = 187)	2.19	1.39–3.46	<0.01
AS+CVD+PAD (n = 29)	0.55	0.13–2.32	0.42
AS+CAD+CVD (n = 71)	1.36	0.58–3.21	0.48
AS+CAD+PAD (n = 138)	1.30	0.68–2.48	0.44
AS+CAD+CVD+PAD (n = 50)	1.74	0.74–4.11	0.20
All-cause death ¶			
AS+CVD (n = 131)	1.46	0.92–2.31	0.11
AS+CAD (n = 485)	1.26	0.92–1.72	0.14
AS+ PAD (n = 187)	1.67	1.16–2.39	<0.01
AS+ CVD + PAD (n = 29)	0.79	0.29–2.18	0.65
AS+ CAD + CVD (n = 71)	1.32	0.71–2.48	0.38
AS+ CAD + PAD (n = 138)	1.62	1.06–2.48	<0.05
AS+ CAD+ CVD + PAD (n = 50)	2.23	1.28–3.87	<0.01
Ischemic stroke §			
AS+CVD (n = 131)	1.03	0.71–1.49	0.89
AS+CAD (n = 485)	1.12	0.92–1.37	0.26
AS+ PAD (n = 187)	1.05	0.79–1.39	0.73
AS+CVD+PAD (n = 29)	1.14	0.63–2.06	0.66
AS+CAD+CVD (n = 71)	1.34	0.89–2.03	0.17
AS+CAD+PAD (n = 138)	1.27	0.94–1.71	0.12
AS+CAD+CVD+PAD (n = 50)	1.34	0.83–2.16	0.23
Hospital readmission £			
AS+CVD (n = 131)	1.03	0.71–1.49	0.89
AS+CAD (n = 485)	1.12	0.92–1.37	0.26
AS+PAD (n = 187)	1.05	0.79–1.39	0.73
AS+CVD +PAD (n = 29)	1.14	0.63–2.06	0.66
AS+CAD +CVD (n = 71)	1.34	0.89–2.03	0.17
AS+CAD+PAD (n = 138)	1.27	0.94–1.71	0.12
AS+CAD+CVD+PAD (n = 50)	1.34	0.83–2.16	0.23
Major or life-threated bleeding @			
AS+CVD (n = 131)	0.48	0.12–1.99	0.31
AS+CAD (n = 485)	1.34	0.79–2.27	0.28
AS+ PAD (n = 187)	1.36	0.66–2.84	0.41
AS+ CVD+PAD (n = 29)	0.73	0.10–5.36	0.76
AS+ CAD+CVD (n = 71)	0.82	0.20–3.44	0.79
AS+ CAD+PAD (n = 138)	1.53	0.71–3.28	0.28
AS+ CAD+CVD+PAD (n = 50)	3.58	1.51–8.51	<0.01

AS = Aortic valve stenosis, CAD = coronary artery disease, CI = confidence interval, CVD = cerebral vascular disease, HR = hazard ratio, PAD = peripheral artery disease, TAVI = transcatheter aortic valve implantation.

#,¶,§,£, and @ adjusted for the same confounders specified in.

Patients with AS with 3 VB diseases (CAD+CVD+PAD; adjusted HR, 2.61; 95% CI, 1.21–5.62; p<0.05) and those with 2 VB diseases (CAD+PAD; adjusted HR, 1.93; 95% CI, 1.21–5.62; p<0.05) had significantly higher adjusted risks of cardiovascular death than those with AS alone. However, AS patients with 1-VB disease of CVD (adjusted HR, 1.91; 95% CI, 1.08–3.38, p<0.05) and those of PAD (adjusted HR, 2.19; 95% CI, 1.39–3.46; p<0.01) had significantly higher risks of non-cardiac death than those with AS only. Accordingly, AS patients with VB diseases (3 VB diseases: CAD+CVD+PAD; adjusted HR, 2.23; 95% CI, 1.28–3.87; p<0.01; 2 VB diseases: CAD+PAD; adjusted HR, 1.62; 95% CI, 1.06–2.48; p<0.05; and 1 VB disease: PAD; adjusted HR, 1.67; 95% CI, 1.16–2.39; p<0.01) had significantly higher adjusted risks of all-cause death than those without VB diseases. Furthermore, patients with 3 VB diseases (CAD+CVD+PAD; adjusted HR, 3.58; 95% CI, 1.51–8.51; p<0.01) had significantly higher risks of major or life-threatening bleeding. As for ischemic stroke and hospital readmission, the significant adjusted hazard ratio was not shown for AS patients with individual VB disease compared with those with AS alone.

Table 5 displays the comparison of causes of death between patients with PVD and those without.

**Table 5:** Comparison of causes of death between patients with and without PVD.

	Patients with PVD (n = 288)	Patients without PVD (n = 1879)	p-value
Procedure mortality	4.5	2.0	<0.01
Cardiovascular mortality			
Worsening heart failure	4.6	1.1	<0.01
Valve-related deaths	1.9	0.4	0.05
Stroke	1.8	1.3	0.33
Myocardial infarction	0	0.1	0.71
Sudden death	1.5	0.8	0.46
Non-coronary vascular death	0	0.1	0.71
Unknown/other deaths	0.4	1	0.50
Non-cardiovascular mortality	10.9	10.1	0.42

Data are presented as Kaplan-Meier percentages (%). P-values were analyzed using the log-rank test. PVD = polyvascular disease.

The procedural mortality (4.5% vs. 2.0%, p<0.01) and worsening HF (4.6% vs. 1.1%, p<0.01) rates were significantly higher in patients with PVD than those without.

The sub-analysis of the causes of death of patients with individual VB disease is shown in Table 6.

**Table 6:** The sub-analysis of the causes of death of patients with individual VB disease for 2 years.

	Patients without PVD (n = 1879)				Patients with PVD (n = 288)				p-value
	AS	AS+	AS+	AS+	AS+	AS+	AS+	AS+	overall
	alone	CVD	CAD	PAD	CVD+PAD	CAD+CVD	CAD+PAD	CAD+CVD+PAD	
	(n = 1076)	(n = 131)	(n = 485)	(n = 187)	(n = 29)	(n = 71)	(n = 138)	(n = 50)	
Procedure mortality	1.6	3.8	1.9	3.3	6.9	2.8	5.1	4.0	0.07
Cardiovascular death									
Worsening heart failure	0.8	1.8	1.5	1.1	0.0	0.0	6.1	9.4	<0.01
Valve related death	0.4	0.0	0.6	0.0	0.0	3.4	1.3	2.1	0.40
Stroke	1.5	0.0	1.3	1.2	0.0	1.5	0.8	5.9	0.29
Myocardial infarction	0.0	0.0	0.2	0.0	0.0	0.0	0.0	0.0	0.86
Sudden death	1.1	0.0	0.4	0.6	0.0	1.4	0.8	6.2	0.77
Non-coronary vascular death	0.0	0.0	0.0	0.7	0.0	0.0	0.0	0.0	0.12
Unknown/other	1.0	0.0	1.0	2.0	0.0	0.0	0.8	0.0	0.93
Non-cardiovascular death	8.0	15.4	10.1	19.3	7.4	10.6	9.0	21.5	<0.01

Data are Kaplan-Meier percentages (%). P-values were analyzed using the log-rank test. AS = Aortic valve stenosis, CAD = coronary artery disease, CI = confidence interval, CVD = cerebral vascular disease, HR = hazard ratio, PAD = peripheral artery disease. Definitions of causes of death were based on Valve Academic Research Consortium-2 criteria. Procedural mortality comprised all-cause mortality within 30 days or during index procedure hospitalization if the postoperative length of stay was longer than 30 days. Valve-related deaths included structural or non-structural valve dysfunction or other valve related adverse events Strokes included ischemic or hemorrhagic strokes. Non-coronary vascular death excluded stroke-related events.

HF worsened the most among patients with 3 VB diseases (CAD+CVD+PAD, 9.4%), followed by those with 2 VB diseases (CAD+PAD, 6.1%), compared with other patients (0%–1.8%, p<0.01). Patients with 3 VB disease (CAD+CVD+PAD) also had the highest rate of non-cardiac death (21.5%), followed by those with PAD only (19.3%) and CVD only (15.4%), as compared with other patients (7.4%–10.6%, p<0.01).

## Discussion

### Main findings

This study investigated the association between PVD and clinical outcomes in patients with transfemoral AS. We have found the following points: (a) patients with PVD had a higher incidence of in-hospital complications, such as AKI and disabling stroke, which resulted in higher rates of procedural mortality, than patients without PVD; (b) furthermore, compared with patients without PVD, those with PVD also had a higher 2-year rate of cardiovascular death, yet the 2-year incidence of non-cardiac death, MI, or ischemic stroke was not associated with PVD; (c) the main cause of 2-year cardiovascular deaths was worsening HF, which resulted in a higher cardiovascular death risk among patients with 3 VB (CAD+CVD+PAD) and 2 VB (CAD+PAD) diseases; and (d) patients with 3 VB diseases had the highest risk of 2-year cardiovascular death, all-cause death, and life-threatening or major bleeding.

### In-hospital complications and procedural mortality

In our study, in-hospital complication such as stroke and AKI was significantly higher in patients with PVD than those without PVD. Vlastra reported that CVD, CAD, and CKD were independent predictors of stroke on the day of TAVI and after 30 days [12]. Moreover, a previous meta-analysis reported that HT, DM, PAD, low EF, and CKD were predictors of AKI after TAVI. HT and DM were also reported to impair renal autoregulation [26]. Patients with CKD are vulnerable to nephrotoxic drugs, renal hypotension, particularly under low EF settings, and inflammatory mediators during TAVI [26]. In our study, these risk factors were frequently observed in patients with PVD, and might be associated with the occurrence of stroke and AKI in hospital after TAVI.

Furthermore, our results may account for the higher burden of atherosclerotic plaque and calcification in patients with PVD, especially in the aorta and valve, compared with that of patients without PVD, which may be disrupted by TAVI. TAVI requires the use of large-sized delivery catheters, balloon valvuloplasty, new valve positioning and implantation, and post-dilation with calcified and plaque-rich aortic wall and valve manipulation. Consequently, the dislodgement and embolization of the crushed calcified native valve and aortic debris occur in the brain and kidney.

Our study has shown that patients with PVD had a higher procedural mortality rate (4.5%) than those without PVD. Previous studies have reported the association between in-hospital complications, such as stroke and AKI, and short-term mortality after TAVI [12].

### Outcomes within two years after TAVI

Patients with PVD had a significantly higher 2-year cardiovascular death and all-cause death rates than those without, which is consistent with the results of previous reports on stable outpatients with acute coronary syndrome (ACS) [6]. However, in our study, there were no differences in the 2-year MI and stroke rates between patients with and without PVD, which is different from the results of previous studies on other atherosclerotic scenarios, wherein patients with PVD had more ischemic events than those without [5, 6]. There may be differences between the high-risk TAVI population and other atherothrombotic middle-aged patients, in whom plaque rupture and thrombotic events are the main causes of cardiovascular events [27]. Accordingly, to the best of our knowledge, this is the first report to demonstrate that PVD has effects on clinical outcomes in a high-risk TAVI cohort.

In a sub-study, patients with AS and 3 VB (CAD+PAD+CVD) and 2 VB (CAD+PAD) diseases had a higher risk of 2-year cardiovascular death than those with AS alone. A recent meta-analysis has demonstrated that CAD did not affect 1-year prognosis after TAVI [10]. Ben-Shoshan has reported that, unlike surgical bypass or aortic valve replacement, extracranial carotid artery stenosis was not associated with worse outcome following TAVI [28]. However, a previous meta-analysis has revealed that the pre-existence of PAD was related to an increased risk of all-cause death in patients undergoing TAVI [11]. In our study, patients with AS with 1 isolated VB disease (CAD, PAD, or CVD) did not have a statistically significant higher risk of cardiovascular death than patients with AS and no VB diseases. A combination of CAD and PAD in AS patients, and even more accumulated atherosclerotic complications, such as 3 VB diseases, was related to a higher risk of cardiovascular death after TAVI. Accordingly, pre-procedural carotid ultrasound screening and ABI for patients undergoing TAVI is meaningful to detect PVD. We previously reported ABI as a prognostic surrogate marker after TAVI [27]. Future studies including a larger number of PVD subsets are required to determine which VB disease primarily provides more risk with AS alone.

In our study, patients with PVD were predominantly male, which had been reported to represent an adverse factor after TAVI in previous studies [29, 30]. Conrotto F reported that factors associated with atherosclerotic disease (i.e. insulin dependent DM, previous stroke, and reduced ejection fraction (EF)) were predictors of midterm mortality in men after TAVI [31]. In our TAVI registry, after adjusting confounders including male, PVD was an independent predictor of cardiovascular mortality for 2 years, suggesting that development of atherosclerotic burden in patients with PVD was related to cardiovascular death irrespective of gender difference.

In this study, the main cause of cardiovascular death was worsening HF. CAD and PAD have been known to be a greater prognostic factor of HF with preserved EF (HFpEF) than CVD [32]. The systemic atherosclerosis mechanism underlying the worsening of HF included the progression of systemic arterial stiffness, vascular endothelial dysfunction, and chronic inflammation [32]. Moreover, LV unloading after TAVI confirmed HF symptom alleviation and LV hypertrophy regression, but this process does not follow a fixed schema, and various factors may affect heart function [33]. In this study, high rates of HT, DM, CKD, and anemia, as well as frailty, in patients with PVD provoked HF. Higher frailty levels are indicative of more frequent rehospitalization in patients with HF and were reported to be mortality predictors following TAVI [14]. In our study, PVD was associated with rehospitalization after TAVI, and in previous studies, the major cause of cardiac rehospitalization was worsening chronic HF (32.6%–46.1%) [24, 33]. Hospitalization itself worsened the performance of activities of daily living, resulting in a reduction in muscle strength, the development of sarcopenia, and HF progression [34].

In contrast, non-cardiovascular death was not different between patients with and without PVD. Patients with AS and only 1 VB disease, i.e.., AS+CVD or AS+PAD, had a significantly higher risk non-cardiac death than those with AS only. Atherosclerosis is considered a chronic inflammatory disease related to an abnormal stimulus of the endothelium that is mostly induced by conditions such as DL, HT, DM, and obesity [35]. These conditions have also been considered as predispositions to cancer [35]. Therefore, the elevated levels of pro-inflammatory systemic molecules, such as interleukins, C-reactive proteins, and tumor necrotic factors, have been associated with not only atherosclerosis, but also non-cardiovascular diseases, including several malignancies and infectious diseases [35]. Even patients with 1 VB disease may have a higher risk of non-cardiac death than patients with no VB disease.

The 2-year incidence of major or life-threatening bleeding tended to be higher in patients with PVD than in those without and independently the highest in patients with 3 VB diseases. The rate of CVD and CKD was also higher in patients with PVD than in patients without. These factors, as reported in a PCI population, resulted in a higher bleeding risk [15].

### Management and treatment

The vascular screening and precise collection of the history of patients are important to diagnose PVD. This is not only for selecting the appropriate treatment approach, but also for performing risk stratification, even in patients undergoing TF-TAVI. The use of cerebral protection devices or switching to alternative approaches may reduce the incidence of TAVI-related stroke among patients with PVD in case that high calcified and plaque burden were found in aortic route [12]. Minimum contrast or the use of iso-osmolor contrast media for patients with PVD in addition to CKD may reduce AKI [36, 37]. Peri-operative blood transfusion has been reported to be an AKI predictor [36]; accordingly, avoiding bleeding complications is crucial in patients with PVD. Duplex echo-guided femoral puncture and the relative selection of sheaths according to the diameter of the femoral artery using computed tomography is useful to avoid bleeding in patients with advanced atherosclerotic PVD [38].

As for medical therapy, different approaches are required for patients with PVD undergoing TAVI from another atherosclerotic cohort such as CAD, PAD, and CVD without AS. Previous studies reported that ACS after TAVI was rare (<5%), and stroke occurring after 30 days was a natural event in elderly TAVI population [4, 12]. PVD was reported to be associated with worsening HF in patients with HFpEF [32]. Thus, for PVD patients who underwent TAVI, according to the results of this study, medical therapy targeting HF may be reasonable. Previous registry data demonstrated that a renin angiotensin blockade, beta-blockers, and statin therapy were associated with favorable clinical outcomes after TAVI [19, 39, 40]. These drugs possess anti-atherosclerotic and HF-treatable effects. Future randomized study is warranted to elucidate these efficacies for TAVI patients.

In our study, patients with PVD, especially those with 3 VB diseases, had a significantly higher 2-year bleeding risk than patients without PVD. The rates of several bleeding risk factors, including HT, CKD, PAD, frailty, and anemia, were higher among patients with PVD than those without [15]. According to these baseline background data, intensive antithrombotic therapy may be avoided for patients with PVD undergoing TAVI. However, a recent meta-analysis of randomized trials of antithrombotic therapy for secondary prevention of cardiovascular events in the cohort of CAD and/or PAD has demonstrated that intensive antithrombotic therapy benefits patients with PVD [13]. Future studies on better antithrombotic strategy for patients with PVD undergoing TAVI may be required to reduce long-term cardiovascular events.

### Limitations

This study has several limitations. First, baseline characteristics were markedly different between both groups, and few patients were in PVD group. Nevertheless, our registry included all-comer patients and monitored the current use of TAVI in real-world settings. This study was a post-hoc investigation. Our findings are hypothesis generating to be further explored in a future randomized trials /prospective registry investigating medical therapy after TAVI with a larger sample size. It can also showcase more specifically which combination of VB disease provides more risk and which optimal medical therapy is effective after TAVI.

Second, recent randomized trials have indicated favorable outcomes of TAVI among low-risk patients whose comorbidities were different from those in our study and early registries [2]. Future studies regarding the clinical impact of PVD on long-term outcomes in these low-intermediate-risk patients using low-profile new generation TAVI devices should be conducted.

## Conclusion

PVD, which is an increase in the number of concomitant atherosclerotic VB diseases, before transfemoral TAVI may increase the incidence of in-hospital complications and the 2-year cardiovascular mortality rate. Given the higher rate of mortality in patients with PVD undergoing TF-TAVI, future studies focusing on medical therapy are needed to reduce long-term cardiovascular events in this high-risk subset.

## Supporting information

S1 File (DOCX)

## References

[1] Smith CR, Leon MB, Mack MJ, Miller DC, Moses JW, Svensson LG, et al. Transcatheter versus surgical aortic-valve replacement in high-risk patients. N Engl J Med. 2011;364:2187–98. doi: 10.1056/NEJMoa1103510 21639811

[2] Mack MJ, Leon MB, Thourani VH, Makkar R, Kodali SK, Russo M, et al. Transcatheter Aortic-Valve Replacement with a Balloon-Expandable Valve in Low-Risk Patients. N Engl J Med. 2019;380:1695–705. doi: 10.1056/NEJMoa1814052 30883058

[3] Popma JJ, Deeb GM, Yakubov SJ, Mumtaz M, Gada H, O’Hair D, et al. Transcatheter Aortic-Valve Replacement with a Self-Expanding Valve in Low-Risk Patients. N Engl J Med. 2019;380:1706–15. doi: 10.1056/NEJMoa1816885 30883053

[4] Mentias A, Desai MY, Saad M, Horwitz PA, Rossen JD, Panaich S, et al. Incidence and Outcomes of Acute Coronary Syndrome After Transcatheter Aortic Valve Replacement. JACC Cardiovascular interventions. 2020;13:938–50. doi: 10.1016/j.jcin.2019.11.027 32061612 PMC7202131

[5] Bhatt DL, Steg PG, Ohman EM, Hirsch AT, Ikeda Y, Mas JL, et al. International prevalence, recognition, and treatment of cardiovascular risk factors in outpatients with atherothrombosis. JAMA. 2006;295:180–9. doi: 10.1001/jama.295.2.180 16403930

[6] Bonaca MP, Gutierrez JA, Cannon C, Giugliano R, Blazing M, Park JG, et al. Polyvascular disease, type 2 diabetes, and long-term vascular risk: a secondary analysis of the IMPROVE-IT trial. The lancet Diabetes & endocrinology. 2018;6:934–43. 30396865 10.1016/S2213-8587(18)30290-0

[7] Morikami Y, Natsuaki M, Morimoto T, Ono K, Nakagawa Y, Furukawa Y, et al. Impact of polyvascular disease on clinical outcomes in patients undergoing coronary revascularization: an observation from the CREDO-Kyoto Registry Cohort-2. Atherosclerosis. 2013;228:426–31. doi: 10.1016/j.atherosclerosis.2013.04.005 23623262

[8] Chen ST, Hellkamp AS, Becker RC, Berkowitz SD, Breithardt G, Fox KAA, et al. Impact of polyvascular disease on patients with atrial fibrillation: Insights from ROCKET AF. American heart journal. 2018;200:102–9. doi: 10.1016/j.ahj.2018.02.013 29898836

[9] Aboyans, Ricco JB, Bartelink MEL, Björck M, Brodmann M, Cohnert T, et al. Editor’s Choice—2017 ESC Guidelines on the Diagnosis and Treatment of Peripheral Arterial Diseases, in collaboration with the European Society for Vascular Surgery (ESVS). European journal of vascular and endovascular surgery: the official journal of the European Society for Vascular Surgery. 2018;55:305–68. doi: 10.1016/j.ejvs.2017.07.018 28851596

[10] D’Ascenzo F, Verardi R, Visconti M, Conrotto F, Scacciatella P, Dziewierz A, et al. Independent impact of extent of coronary artery disease and percutaneous revascularisation on 30-day and one-year mortality after TAVI: a meta-analysis of adjusted observational results. EuroIntervention: journal of EuroPCR in collaboration with the Working Group on Interventional Cardiology of the European Society of Cardiology. 2018;14:e1169–e77. doi: 10.4244/EIJ-D-18-00098 30082258

[11] Ueshima D, Barioli A, Nai Fovino L, D’Amico G, Fabris T, Brener SJ, et al. The impact of pre-existing peripheral artery disease on transcatheter aortic valve implantation outcomes: A systematic review and meta-analysis. Catheterization and cardiovascular interventions: official journal of the Society for Cardiac Angiography & Interventions. 2020;95:993–1000. doi: 10.1002/ccd.28335 31099970

[12] Vlastra W, Jimenez-Quevedo P, Tchetche D, Chandrasekhar J, de Brito FS Jr., Barbanti M, et al. Predictors, Incidence, and Outcomes of Patients Undergoing Transfemoral Transcatheter Aortic Valve Implantation Complicated by Stroke. Circulation Cardiovascular interventions. 2019;12:e007546. doi: 10.1161/CIRCINTERVENTIONS.118.007546 30871358

[13] Weissler EH, Jones WS, Desormais I, Debus S, Mazzolai L, Espinola-Klein C, et al. Polyvascular disease: A narrative review of current evidence and a consideration of the role of antithrombotic therapy. Atherosclerosis. 2020;315:10–7. doi: 10.1016/j.atherosclerosis.2020.11.001 33190107

[14] Shimura T, Yamamoto M, Kano S, Kagase A, Kodama A, Koyama Y, et al. Impact of the Clinical Frailty Scale on Outcomes After Transcatheter Aortic Valve Replacement. Circulation. 2017. 10.1161/CIRCULATIONAHA.116.025630 28302751

[15] Urban P, Mehran R, Colleran R, Angiolillo DJ, Byrne RA, Capodanno D, et al. Defining high bleeding risk in patients undergoing percutaneous coronary intervention: a consensus document from the Academic Research Consortium for High Bleeding Risk. European heart journal. 2019;40:2632–53. doi: 10.1093/eurheartj/ehz372 31116395 PMC6736433

[16] Kappetein AP, Head SJ, Généreux P, Piazza N, van Mieghem NM, Blackstone EH, et al. Updated standardized endpoint definitions for transcatheter aortic valve implantation: the Valve Academic Research Consortium-2 consensus document. European heart journal. 2012;33:2403–18. doi: 10.1093/eurheartj/ehs255 23026477

[17] Yamamoto M, Otsuka T, Shimura T, Yamaguchi R, Adachi Y, Kagase A, et al. Clinical risk model for predicting 1-year mortality after transcatheter aortic valve replacement. Catheterization and cardiovascular interventions: official journal of the Society for Cardiac Angiography & Interventions. 2021;97:E544–e51. 10.1002/ccd.29130 PMC7983930 32729657

[18] Ito S, Taniguchi T, Shirai S, Ando K, Watanabe Y, Yamamoto M, et al. The Impact of Baseline Thrombocytopenia on Late Bleeding and Mortality After Transcatheter Aortic Valve Implantation (From the Japanese Multicenter OCEAN-TAVI Registry). The American journal of cardiology. 2021;141:86–92. doi: 10.1016/j.amjcard.2020.11.017 33220320

[19] Ochiai T, Saito S, Yamanaka F, Shishido K, Tanaka Y, Yamabe T, et al. Renin-angiotensin system blockade therapy after transcatheter aortic valve implantation. Heart (British Cardiac Society). 2018;104:644–51. doi: 10.1136/heartjnl-2017-311738 28986405

[20] Masuyama T, Tsujino T, Origasa H, Yamamoto K, Akasaka T, Hirano Y, et al. Superiority of long-acting to short-acting loop diuretics in the treatment of congestive heart failure. Circulation journal: official journal of the Japanese Circulation Society. 2012;76:833–42. doi: 10.1253/circj.cj-11-1500 22451450

[21] Takagi H, Hari Y, Kawai N, Ando T. Impact of concurrent tricuspid regurgitation on mortality after transcatheter aortic-valve implantation. Catheterization and cardiovascular interventions: official journal of the Society for Cardiac Angiography & Interventions. 2019;93:946–53. doi: 10.1002/ccd.27948 30474201

[22] Koyama Y, Yamamoto M, Kagase A, Tsujimoto S, Kano S, Shimura T, et al. Prognostic impact and periprocedural complications of chronic steroid therapy in patients following transcatheter aortic valve replacement: Propensity-matched analysis from the Japanese OCEAN registry. Catheterization and cardiovascular interventions: official journal of the Society for Cardiac Angiography & Interventions. 2020;95:793–802. doi: 10.1002/ccd.28332 31112003

[23] Giordana F, D’Ascenzo F, Nijhoff F, Moretti C, D’Amico M, Biondi Zoccai G, et al. Meta-analysis of predictors of all-cause mortality after transcatheter aortic valve implantation. The American journal of cardiology. 2014;114:1447–55. doi: 10.1016/j.amjcard.2014.07.081 25217456

[24] Arai T, Yashima F, Yanagisawa R, Tanaka M, Shimizu H, Fukuda K, et al. Hospital readmission following transcatheter aortic valve implantation in the real world. International journal of cardiology. 2018;269:56–60. doi: 10.1016/j.ijcard.2018.07.073 30064926

[25] Guedeney P, Huchet F, Manigold T, Rouanet S, Balagny P, Leprince P, et al. Incidence of, risk factors for and impact of readmission for heart failure after successful transcatheter aortic valve implantation. Archives of cardiovascular diseases. 2019;112:765–72. doi: 10.1016/j.acvd.2019.09.008 31759916

[26] Liao YB, Deng XX, Meng Y, Zhao ZG, Xiong TY, Meng XJ, et al. Predictors and outcome of acute kidney injury after transcatheter aortic valve implantation: a systematic review and meta-analysis. EuroIntervention: journal of EuroPCR in collaboration with the Working Group on Interventional Cardiology of the European Society of Cardiology. 2017;12:2067–74. doi: 10.4244/EIJ-D-15-00254 27890858

[27] Yamawaki M, Araki M, Ito T, Honda Y, Tokuda T, Ito Y, et al. Ankle-brachial pressure index as a predictor of the 2-year outcome after transcatheter aortic valve replacement: data from the Japanese OCEAN-TAVI Registry. Heart and vessels. 2018;33:640–50. doi: 10.1007/s00380-017-1096-y 29230568

[28] Ben-Shoshan J, Zahler D, Steinvil A, Banai S, Keren G, Bornstein NM, et al. Extracranial carotid artery stenosis and outcomes of patients undergoing transcatheter aortic valve replacement. International journal of cardiology. 2017;227:278–83. doi: 10.1016/j.ijcard.2016.11.107 27839800

[29] Hayashida K, Morice MC, Chevalier B, Hovasse T, Romano M, Garot P, et al. Sex-related differences in clinical presentation and outcome of transcatheter aortic valve implantation for severe aortic stenosis. J Am Coll Cardiol. 2012;59:566–71. doi: 10.1016/j.jacc.2011.10.877 22300690

[30] Zhao ZG, Liao YB, Peng Y, Chai H, Liu W, Li Q, et al. Sex-related differences in outcomes after transcatheter aortic valve implantation: a systematic review and meta-analysis. Circulation Cardiovascular interventions. 2013;6:543–51. doi: 10.1161/CIRCINTERVENTIONS.113.000529 24065446

[31] Conrotto F, D’Ascenzo F, Salizzoni S, Presbitero P, Agostoni P, Tamburino C, et al. A gender based analysis of predictors of all cause death after transcatheter aortic valve implantation. The American journal of cardiology. 2014;114:1269–74. doi: 10.1016/j.amjcard.2014.07.053 25159239

[32] Fujisue K, Tokitsu T, Yamamoto E, Sueta D, Takae M, Nishihara T, et al. Prognostic significance of polyvascular disease in heart failure with preserved left ventricular ejection fraction. Medicine. 2019;98:e15959. doi: 10.1097/MD.0000000000015959 31305390 PMC6641821

[33] Franzone A, Pilgrim T, Arnold N, Heg D, Langhammer B, Piccolo R, et al. Rates and predictors of hospital readmission after transcatheter aortic valve implantation. European heart journal. 2017;38:2211–7. doi: 10.1093/eurheartj/ehx182 28430920

[34] Takabayashi K, Ikuta A, Okazaki Y, Ogami M, Iwatsu K, Matsumura K, et al. Clinical Characteristics and Social Frailty of Super-Elderly Patients With Heart Failure- The Kitakawachi Clinical Background and Outcome of Heart Failure Registry. Circulation journal: official journal of the Japanese Circulation Society. 2016;81:69–76. doi: 10.1253/circj.CJ-16-0914 27904019

[35] Tapia-Vieyra JV, Delgado-Coello B, Mas-Oliva J. Atherosclerosis and Cancer; A Resemblance with Far-reaching Implications. Archives of medical research. 2017;48:12–26. doi: 10.1016/j.arcmed.2017.03.005 28577865

[36] Bagur R, Webb JG, Nietlispach F, Dumont E, De Larochelliere R, Doyle D, et al. Acute kidney injury following transcatheter aortic valve implantation: predictive factors, prognostic value, and comparison with surgical aortic valve replacement. European heart journal. 2010;31:865–74. doi: 10.1093/eurheartj/ehp552 20037180 PMC2848323

[37] Iacovelli F, Pignatelli A, Cafaro A, Stabile E, Salemme L, Cioppa A, et al. Impact of contrast medium osmolality on the risk of acute kidney injury after transcatheter aortic valve implantation: insights from the Magna Graecia TAVI registry. International journal of cardiology. 2021;329:56–62. doi: 10.1016/j.ijcard.2020.12.049 33359334

[38] Hayashida K, Lefevre T, Chevalier B, Hovasse T, Romano M, Garot P, et al. Transfemoral aortic valve implantation new criteria to predict vascular complications. JACC Cardiovascular interventions. 2011;4:851–8. doi: 10.1016/j.jcin.2011.03.019 21851897

[39] Saito T, Yoshijima N, Hase H, Yashima F, Tsuruta H, Shimizu H, et al. Impact of beta blockers on patients undergoing transcatheter aortic valve replacement: the OCEAN-TAVI registry. Open heart. 2020;7. doi: 10.1136/openhrt-2020-001269 32641381 PMC7342827

[40] Takagi H, Hari Y, Nakashima K, Kuno T, Ando T. Meta-Analysis for Impact of Statin on Mortality After Transcatheter Aortic Valve Implantation. The American journal of cardiology. 2019;124:920–5. doi: 10.1016/j.amjcard.2019.05.069 31326076

