# Supplementary material for: Influence of polyvascular disease on clinical outcome in patients undergoing transcatheter aortic valve implantation via transfemoral access
Source: PLoS One. 2021 Dec 2;16(12):e0260385. doi: 10.1371/journal.pone.0260385 (PMC8638934; doi:10.1371/journal.pone.0260385)
Supplement: S1 File — (DOCX) [file pone.0260385.s001.docx]

The OCEAN-TAVI registry is supported by Edwards Lifesciences(<http://www.edwards.com>), Medtronic(<https://www.medtronic.com>), and the Daiichi-Sankyo company(<https://www.daiichisankyo.com>). The funders had no role in study design, data, collection and analysis, decision to publish, or preparing of the manuscript.
